# Evaluation of the camel milk amelioration, the oxidative stress, fertility and mutagenicity of male albino rats exposed to lead acetate, fipronil, and their mixture

**DOI:** 10.1002/fsn3.3663

**Published:** 2024-02-04

**Authors:** Yasmin E. Abdel‐Mobdy, Ahmed E. Abdel‐Mobdy, Ammar AL‐Farga, Faisal Aqlan

**Affiliations:** ^1^ Entomology and Pesticide Department Faculty of Agriculture Cairo University Giza Egypt; ^2^ Dairy Science Department Faculty of Agriculture Cairo University Giza Egypt; ^3^ Department of Biochemistry College of Science University of Jeddah Jeddah Saudi Arabia; ^4^ Department of Chemistry College of Sciences Ibb University Ibb Governorate Yemen

**Keywords:** camel milk, chromosomal aberration, fipronil, histopathology, lead, oxidative stress, sperm

## Abstract

Lead is considered a common old chronic toxicant around the world due to expanded environmental pollution, it is likely an inevitable contaminant in food, dairy products, air etc. Also, fipronil is a wide‐ranging effective N‐phenyl pyrazole insecticide which used commonly in agriculture and public health insect control, but until now no adequate data are available on the oxidative stress, cytotoxicity, and mutagenic influence of fipronil and lead or their mixture subchronic exposure. Both xenobiotics (lead and fipronil) exert a harmful impact on reproduction, prompting the exploration of various foods for functional protection. The present study investigated the effects of camel milk treatments on reproductive problems caused by lead acetate with or without mixing with fipronil in male albino rats. Liver oxidative stress, testicular relative weight, sperm analysis, investigation of chromosomal aberration, and histopathological examination of testis were performed. The results showed that the oxidative stress was elevated in rats treated with fipronil, lead acetate, and their mixture, which were reduced through camel milk treatments. Sperm counts were decreased significantly in lead and/or fipronil exposure but significantly elevated with camel milk intoxicated treated. Sperm morphological abnormalities and chromosomal aberrations in intoxicated groups were reduced significantly in camel milk‐treated animals relative to untreated intoxicated groups. Testicular histopathological results showed moderate common degeneration of seminiferous tubules in lead and/or fipronil‐intoxicated rats which were ameliorated by camel milk treatments. Generally, it can be concluded that lead and fipronil together in a mixture resulted in or induced severe reproductive problems and oxidative stress over lead or fipronil alone. Camel milk treatment significantly decreased the harmful oxidative stress in reproductive as well as the mutagenicity disorder associated with lead and fipronil exposure in male albino rats.

## Graphical abstract

Generally, it can be concluded that lead and fipronil together in a mixture resulted in or induced severe reproductive problems and oxidative stress more than lead or fipronil alone. Camel milk treatment significantly decreased the harmful oxidative stress in reproductive as well as the mutagenicity disorder associated with lead and fipronil exposure in male albino rats.

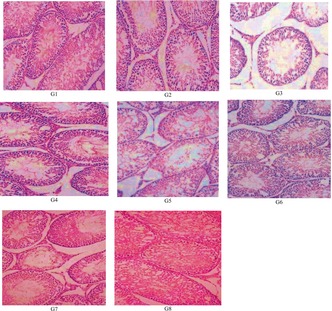

## 1 INTRODUCTION

Fipronil is considered to be an insecticide which belongs to the phenylpyrazole chemical group. It is a broad‐spectrum insecticide commonly used in Egypt to control agricultural, veterinary, and public health insects (Noaishi et al., 2021). Fipronil usage considerably increased as it is used as a replacement for traditional insecticides such as organophosphates and pyrethroids pesticides (Abdel‐Mobdy et al., 2023). Fipronil causes hyperexcitation resulting from its binding affinity to the Gamma‐aminobutyric acid receptors causing a blockage of glutamate‐gated chloride channels (Kandil et al., 2020). Fipronil induction into animals decreased the antioxidant system including the activities of superoxide dismutase (SOD), catalase, and glutathione s‐transferase (GST) as well as glutathione (GSH) reduced levels (Kartheek & David, 2018; Mossa et al., 2015; Noaisha et al., 2021). Fipronil also induced genotoxic and mutagenic potential as well as chromosomal aberrations (Badgujar et al., 2017; Noaisha & Abd Alhafez, 2017; Noaisha et al., 2021). However, no or very little data are available in the literature about the influences of fipronil formulations.

Lead is counted to be a highly toxic metal and its widespread use has caused high environmental pollution and health problems in many parts of the world. Unlike some metals, such as zinc and manganese, lead does not play any biological functions; instead, it disturbs various organisms' physiological processes (Underwood, 2012). The harmful effects of lead depend on its chemical forms inducted into animals, route of induction, and frequency with duration inducting it into animals (Abdel‐Mobdy et al., 2023; Abdel‐Rahim et al., 2014). Also, it has been related to different forms of cancer in humans (Pitot & Dragan, 1996).

The lead induction including ingestion affected the immune system such as IgG, IgA, and IgM levels in the animal's blood and reproductive parameters like sperm examination which showed frequent abnormalities of sperms and also decreased sperm count (Ercal et al., 2000; Ibrahim et al., 2012). The antioxidative system of animals such as SOD and GPx was found to have an overwhelming part in lead exposure (Kang et al., 2004). Under lead exposure, a significant increase in oxidative harms based on malondialdehyde (MDA) levels was observed in peripheral blood mononuclear cells (PMCs) (Ercal et al., 2000), which means that lead oral exposure causes alterations in biochemical parameters and testicular, epididymal, and pituitary function (Allouche et al., 2009). Otherwise, male Wister rats showed lead accumulation with a verbal harmful impact in kidneys, stomach, blood, heart, spleen, and liver. The lead retention and organ dissemination change depending upon the course of lead administration (Nwokocha et al., 2012).

Many investigations consider the beneficial attributes of camel milk, it has potential therapeutic characteristics, such as antihypertensive, antidiabetic, antioxidant, anticarcinogenic etc. It contains malady‐battling immunoglobulin, which are small in size, permitting infiltration of antigens and boosting the performance of the immune system. Camel milk is rich in minerals, vitamin C, and protective protein (Hamed et al., 2011; Yadav et al., 2015). Also, camel milk encompasses a high exceptional concentration of mono‐ and polyunsaturated fatty acids, lactoferrin, serum albumin, vitamins C and E, immunoglobulins, lysozyme, iron, and manganese in addition to the hormone insulin (Kaskous, 2016; Yadav et al., 2015).

The present study investigated the oxidative stress of spermatogenic injuries, sperm abnormalities, and chromosomal aberration produced by lead acetate, fipronil, and their mixture and assessed the effectiveness of camel milk intake versus those harmful effects of lead and fipronil utilizing biochemical and histological examinations.

## 2 MATERIALS AND METHODS

### 2.1 Camel Milk

Camel milk was collected from the camel milk farm at Marsa Matroh station. Animal Production Research Institute, Agricultural Research Center, Giza, Egypt. Milk samples were kept in cooled boxes until transported to the laboratory.

### 2.2 LD_50_

The acute oral toxicity LD_50_ of lead acetate and fipronil formulate (Coach 20% SC, manufactured by Starchem) was determined according to the method of Weil (1952). The LD_50_ values were 5050 and 118 mg/kg albino male rats' body weight for lead acetate and fipronil formulation, respectively. Lead acetate was obtained from Sigma Chemical Co. Egypt, but the fipronil formulation (Coach 20% SC) was obtained from the Center of Agriculture Pesticide Laboratory ARC.

### 2.3 Test animal

The male albino rats obtained from the National Research Center were housed in plastic cages (two rats per cage) and acclimated for 2 weeks. The temperature and relative humidity were adjusted at 25–72°C and 50%–60%, respectively. The experimental rats were fed a normal diet and had free access to water. These studies were approved by the Institutional Animal Care and Use Committee, faculty of veterinary medicine, Cairo University (Vet Cu 011020224) and were carried out in accordance with the guidelines of care and use of Laboratory Animals stated by the National Institutes of Health, USPHS. A normal control diet according to the AIN‐76 was prepared for all experimental animals.

Forty‐eight male albino rats were randomly assigned to eight groups (six rats each). G1 was the normal healthy control. G2, G4, G6, and G8 were normal healthy groups ingested by 2 mL of camel milk/100 g body weight. G3 and G4 ingested by 1/30 LD_50_ of fipronil formulation. G5 and G6 ingested by 1/30 LD_50_ of lead acetate. G7 and G8 ingested by 1/60 LD_50_ of lead acetate and 1/60 LD_50_ of fipronil formulation. G1, G3, G5, and G7 were used as normal fipronil, lead acetate, and fipronil + lead acetate controls, respectively, but the other groups were used as treated intoxicated groups. The animals were freely allowed to access the tap water and were fed on basal diet composed of 15% casein, 10% corn oil, 5% cellulose, 4% salt mixture, 1% vitamin mixture, and 65% starch according to the AIN‐76. All rats were weighed at the beginning and end of the experimental period (3 months). Animals were euthanized after months; serum and fresh organ samples were collected.

### 2.4 Biochemical examination

The biochemical examination was done by commercial enzymatic kits (obtained from Biodiagnostic) which were used for the determinations of liver MAD and GSH contents as well as SOD, CAT, and GST activities according to Ohakawa et al. (1979), Beutler (1963), Nishikimi et al. (1972), Aebi (1984), and Habig et al. (1974), respectively.

Body weight and relative testicular weight were determined by following the procedure of animals being weighed at the beginning of experiment to record their body weight. The testis of each rat was excised, blotted, and weighed, then the organ weight/body weight ratio was calculated by following the equation: testis weight/initial body weight × 100, and the animals were sacrificed after 35 days of the first treatment for sperms.

*Sperm analysis* was performed in euthanized rats. For shape examination, the epididymis was excised and mined in about 10 mL of physiological saline, dispersed, and filtered to exclude large tissue fragments. Smears were prepared after staining the sperms with Eosin Y (aqueous) according to the method of Wyrobek (1978) and Wyrobek et al. (1990). At least 4000 sperms per group were assessed for morphological abnormalities. Epididymal sperm count was also determined by a hemocytometer.

### 2.5 Chromosomal analysis

The chromosomal analysis was performed and the rats were sacrificed after 15 days of the first induction, and then e studied for chromosomal aberration analysis. Fewer bones were collected from the euthanized animals and the bone marrow was prepared according to the method of Yosida et al. (1971) and stained with phosphate buffered solution. Chromosomal aberrations such as chromosomes of chromatid gap, break, deletion, and centromeric attenuation were recorded in at roast 50 well metaphase spread for each animal. The mitotic activity of bone marrow cells was determined for each treated and control animal. It is expressed by the mitotic CMI: number of dividing cells in 10,000 cells.

### 2.6 Histopathology

The histopathology was done on tissue specimens from the testis of rats at the end of the experiment, which were fixed in 10% neutral buffered formalin. Specimens were then processed, embedded in paraffin, sectioned, (3–4 mm), and stained by hematoxylin and eosin stain (Bancroft & Layton, 2012) Tissue slides were examined by light microscopy and photographed using a digital camera (Olympus XC 30). The epithelium thickness lining seminiferous tubules were determined using TS view software from the basement membrane to the lumen in 10 tubules/testis at an angle of 90°C to calculate the mean of epithelial thickness/rat.

The histopathological changes of spermatogenesis in 10 seminiferous tubules were graded using Johnsen's score on a scale from 1 to 10 (Abdelatty et al., 2020; Johnsen, 1970). Seminiferous tubules showing no seminiferous epithelium is scored 1, presence of Sertoli cells only and no germinal cells are scored 2, presence of spermatogonia only was scored 3, few spermatocytes with no spermatozoa of spermatids was scored 4, many spermatocytes with no spermatozoa of spermatids with no spermatozoa or spermatids with no spermatozoa and no late spermatids are scored 6, many early spermatids with no spermatozoa and no late spermatids are scored 7, few late spermatids and less than five spermatozoa per tubule are scored 8, many late spermatids, disorganized epithelium indicating slightly impaired spermatogenesis are scored 9, and full spermatogenesis and perfect tubules are scored 10.

### 2.7 Statistical analysis

The statistical analysis was done and the data were tested for homogeneity of variances and analyzed by one‐way ANOVA in statistical package SPSS version 20.0 (SPSS Inc.) followed by post hoc tests (Duncan's tests). A significant was considered at *p* ≤ .05. The Johnsen's score of spermatogenesis was analyzed by using a nonparametric Kruskal–Wallis test to detect significance at *p* ≤ .05. Significant parameters were analyzed by the Mann–Whitney test to show significant between the experimental animal groups.

## 3 RESULTS AND DISCUSSION

Lead and fipronil are considered as an environment of pollutant but their mixture is more toxic than that of lead or fipronil alone. The results represented in Table 1 show the effect of lead acetate and fipronil in addition to their mixture on the liver peroxidation (MAD) and antioxidant parameters such as the content of GSH and the efficiency of GAT, SOD, and GST antioxidative enzyme of liver tissue relative to the different controls (normal, fipronil, and their mixture control).

**TABLE 1:** Liver tissue peroxidation (MAD) and antioxidative parameter (GSH, SOD, CAT, and GST) of the experimental rats.

Groups	MAD content		GSH content		SOD activity		CAT activity		GST activity	
	N mol/g	%	M mol/g	%	μ/g	%	μ/g	%	M mol/g	%
G1	6.401 ± 0.411 e	100	0.422 ± 0.031 a	100	87.00 ± 4.13 a	100	160.00 ± 9.26 a	100	6.14 ± 0.42 a	100
G2	6.372 ± 0.396 e	100	0.430 ± 0.029 a	102	79.77 ± 3.96 a	102	162.01 ± 10.00 a	101	6.20 ± 0.41 a	101
G3	10.212 ± 0.721 b	160	0.221 ± 0.016 c	52	48.11 ± 2.82 b	62	98.00 ± 6.76 bc	61	4.13 ± 0.23 c	67
G4	8.133 ± 0.521 d	127	0.322 ± 0.023 b	76	53.00 ± 3.53 b	68	110.21 ± 6.62 b	69	5.00 ± 0.29 b	81
G5	9.984 ± 0.666 bc	156	0.217 ± 0.017 c	51	50.00 ± 3.71 b	64	102.22 ± 6.71 b	64	4.24 ± 0.31 c	69
G6	8.220 ± 0.601 d	128	0.296 ± 0.020 b	70	55.12 ± 4.00 b	71	112.13 ± 7.16 b	70	4.89 ± 0.28 b	80
G7	11.111 ± 0.100 a	174	0.199 ± 0.014 c	47	36.12 ± 2.96 c	46	87.92 ± 7.11 c	55	3.98 ± 0.27 c	65
G8	9.322 ± 0.830 c	146	0.282 ± 0.021 b	67	51.11 ± 4.01 b	66	99.79 ± 7.77 bc	62	4.11 ± 0.28 c	67

*Note*: % at normal healthy control. All values are represented as mean ± SD. Means with different letters are significantly different at *p* ≤ .05 in the same column.

### 3.1 Biochemical examination

Lipid peroxidation was significantly increased but GSH level in liver tissue was significantly diminished in all intoxicated controls or the treated ones compared to the healthy normal control (groups G1 and G2), with highest elevation in G7 which considered as fipronil + lead acetate intoxicated control, followed by G3 receiving fipronil and G5 receiving lead acetate without treatments (fipronil and lead acetate intoxicated control, respectively).

The intoxicated animal groups treated with camel milk (G4, G6, and G8) presented a significant amelioration and decrease in MDA level but increase in GSH content compared to their peers. In addition, the antioxidant enzyme activity of liver tissue, such as SOD, CAT, and GST, was significantly decreased in all intoxicated groups either with or without camel milk treatment compared to those of normal healthy control (G1). These toxic effects of the lead acetate, fipronil, and their mixture were reduced under the treatments with camel milk, in which the antioxidative enzyme activity was improved and significantly restored in part for the intoxicated groups receiving camel milk treatments versus lead and fipronil toxicity.

The data of spermatogenic damage of the experiment rats are presented in Table 2. The testis weight/body weight of rats was significantly reduced in all intoxicated groups with or without camel milk treatments compared with normal healthy control (G1 and G2), which means that the induction of fipronil or lead acetate or their mixture resulted in a harmful leanness as emaciation in the testes tissue for intoxicated control groups (G3, G5, and G7). In addition, the same direction was seen in the sperm counts for intoxicated control groups. The count of sperm was significantly decreased in lead and fipronil mixture control treatment (G7), lead control groups (G5), and fipronil control group (G3), respectively, relative to that on the healthy normal controls (G1 and G2). The sperm count was enhanced and elevated significantly in the intoxicated groups exposed to camel milk (G4, G6, and G8) relative to their intoxicated untreated controls (G3, G5, and G8). These findings confirmed each other that the present toxicity of lead and fipronil on testis was ameliorated under the treatments with camel milk in G4, G6, and G8, but the results were lower than both normal healthy groups (G1 and G2).

**TABLE 2:** Spermatogenic damage of experimental rats.

Groups	Body weight (BW)			Testis weight (TW)		TW/BW ratio		Sperm count	
	IBW/g	FBW/g	%	TW/g	%	g/100 (BW)	%	Value × $10^{6}$	%
G1	120.00 ± 2.00 a	303.45 ± 8.11 a	100	3.76 ± 0.18 a	100	1.24 ± 0.061 a	100	51.99 ± 3.11 a	100
G2	118.43 ± 3.55 a	312.01 ± 13.16 a	103	3.84 ± 0.26 a	102	1.23 ± 0.083 a	99	52.01 ± 3.61 a	100
G3	123.12 ± 2.33 a	185.33 ± 15.00 c	61	1.87 ± 0.13 c	50	1.01 ± 0.071 b	81	30.00 ± 2.11 c	58
G4	121.77 ± 2.11 a	255.42 ± 12.91 b	84	2.84 ± 0.14 b	76	1.11 ± 0.055 ab	89	38.00 ± 2,46 b	73
G5	119.81 ± 2.92 a	191.32 ± 11.11 c	63	1.824 ± 0.10 c	48	0.95 ± 0.043 b	77	31.11 ± 2.13 c	60
G6	122.25 ± 3.21 a	248.24 ± 12.63 b	81	2.81 ± 0.16 b	75	1.13 ± 0.066 ab	91	38.14 ± 2.01 b	73
G7	120.00 ± 2.22 a	114.12 ± 6.66 d	38	1.12 ± 0.06 d	30	0.99 ± 0.052 b	80	27.77 ± 2.00 c	53
G8	144.41 ± 1.99 a	135.70 ± 10.13 d	45	1.48 ± 0.07 cd	39	1.09 ± 0.053 b	79	37.41 ± 2.41 b	79

*Note*: % at normal healthy control. Every value is shown as mean ± SD. Means with various letters differ significantly at *p* ≤ .05 in the same column.

Abbreviations: IBW, initial body weight; FBW, final body weight.

Therefore, the findings of spermatocyte analysis for the structural and numerical abnormalities of the examined intoxicated animal with or without camel milk treatments are presented in Table 3, which observed the frequencies of sperm abnormality/4000 sperm tested in the experimental rats. The data showed more frequent abnormalities of sperms within the tail and head than normal healthy controls (G1 and G2) and camel milk‐treated groups (G4, G6, and G8). The oral ingestion of camel milk into intoxicated rats decreased the percentage of abnormal sperms, but these frequencies are still significantly exceeding relative to those of normal healthy control animals (G1 and G2). The total number of abnormal sperms varied between the other intoxicated controls and the intoxicated rats treated with camel milk.

**TABLE 3:** Frequencies of sperm abnormality/4000 sperm in each of the different experimental groups.

Groups	No. of total sperm examined	Abnormal sperm		Types of sperm abnormalities (TSHA)							Non‐TSHA	
		No.	4000	Amorphous	Banana shape	Without hook	Big	Small	Total no.	%	Total no.	%
G1	4000	76 ± 4.44	1.9	20 ± 1.22	16 ± 1.01	20 ± 1.12	1 ± 0.01	11 ± 0.7	68 ± 4.20 f	1.7	8	0.2
G2	4000	72 ± 5.13	1.8	18 ± 1.00	15 ± 1.01	19 ± 1.11	0.00	10 ± 0.6	44 ± 3.30 f	1.1	28	0.7
G3	4000	1151 ± 55	29	401 ± 30.11	250 ± 17	99 ± 5.6	131 ± 7.3	101 ± 6.2	781 ± 41.20 c	19.53	370	9.25
G4	4000	500 ± 33	13	129 ± 7.31	70 ± 4.12	65 ± 3.0	30 ± 1.8	97 ± 5.6	391 ± 27.3 e	9.78	109	2.73
G5	4000	1111 ± 65	28	391 ± 20.2	243 ± 14.3	88 ± 5.2	122 ± 7.4	90 ± 5.1	934 ± 52.0 b	23.25	177	4.43
G6	4000	451 ± 33	11	125 ± 7.21	46 ± 2.74	61 ± 2.8	55 ± 3.2	138 ± 7.4	425 ± 25.9 e	10.63	26	0.65
G7	4000	1201 ± 70	30	412 ± 26.6	256 ± 13.33	110 ± 6.6	137 ± 7.1	111 ± 6.1	1026 ± 60.2 a	25.65	175	4.38
G8	4000	711 ± 41	18	200 ± 12.12	70 ± 4.12	125 ± 7.1	100 ± 6.1	120 ± 7.1	615 ± 35.99 d	15.38	96	2.40

*Note*: % at normal healthy control. Every value is shown as mean ± SD. Means with various letters differ significantly at *p* ≤ 0.05 in the same column.

Anomalies of sperm head were clearly elevated in groups G7, G5, and G3, respectively, relative to normal healthy control groups (G1 and G2), which these were reduced in the intoxicated groups treated with camel milk (G8, G6, and G4, respectively), but the results also were higher than those of normal healthy controls (G1 and G2).

### 3.2 Chromosomal aberrations

In the case of chromosomal aberrations, the cytogenic results observed frequencies of structural chromosomal aberrations, numerical chromosomal aberrations, and mitotic activity induced by both toxicants (lead and fipronil) and the modulatory function of camel milk treatment in bone marrow cells of albino rat male are presented in Table 4. The gabs, deletions, breaks, and centromere chromosomal aberration of chromatid were the main kinds of chromosomal aberrations and the ingestion of lead acetate or fipronil and their mixture produced significant elevation in the chromosomal aberration compared with normal healthy control (G1 and G2). The treatment of camel milk against the intoxicated animal with lead or fipronil or their mixture groups attenuated the harmfulness of the three toxicants, which improved the disturbed results of the present parameters, which were slightly lower than those of three intoxicated controls (G3, G5, and G7).

**TABLE 4:** Structural chromosomal aberration induced in male bone marrow cells of the different experimental groups.

Groups	Types of chromosomal aberration				Total chromosomal aberration (T. aber)	Total aberration excluding gaps	Mitotic activity	
	Gap	Deletion	Break	Centromeric			No of examined cells	MI
G1	4.79 ± 0.26	0.81 ± 0.05	0.62 ± 0.03	0.60 ± 0.04	6.82 ± 0.33 d	2.03 ± 0.14 d	10,000	16.71 ± 1.11 d
G2	3.98 ± 0.24	0.90 ± 0.04	0.65 ± 0.04	0.61 ± 0.03	6.14 ± 0.41 d	2.16 ± 0.16 d	10,000	16.82 ± 1.02 a
G3	11.31 ± 0.65	3.21 ± 0.20	2.61 ± 0.16	2.62 ± 0.11	19.75 ± 1.11 b	8.44 ± 0.66 b	10,000	9.50 ± 0.87 c
G4	6.00 ± 0.41	2.62 ± 0.12	1.60 ± 0.11	2.41 ± 0.10	12.63 ± 0.79 c	6.63 ± 0.42 c	10,000	12.54 ± 0.96 b
G5	11.62 ± 0.83	3.10 ± 0.17	2.58 ± 0.17	2.56 ± 0.16	19.86 ± 1.00 b	8.24 ± 0.68 b	10,000	9.54 ± 0.54 c
G6	5.62 ± 0.32	1.99 ± 0.11	1.80 ± 0.11	2.18 ± 0.13	11.59 ± 0.66 c	5.97 ± 0.37 c	10,000	13.11 ± 0.71 b
G7	12.00 ± 0.83	3.31 ± 0.21	3.19 ± 0.21	3.18 ± 0.18	21.68 ± 1.41 a	9.68 ± 0.71 a	10,000	9.44 ± 0.57 c
G8	7.11 ± 0.79	3.96 ± 0.20	1.82 ± 0.17	1.76 ± 0.11	13.76 ± 0.81 c	6.65 ± 0.42 c	10,000	11.94 ± 0.81 bc

*Note*: % at normal healthy control. Every value is shown as mean ± SD. Means with various letters differ significantly at *p* ≤ .05 in the same column.

Also, the chromosomal aberration frequencies of intoxicated control animals were significantly affected. It was observed that treatments with camel milk against the xenobiotics harmful lead and fipronil in groups (G4, G6, and G7) reduced those chromosomal aberration frequencies. In the case of mitotic activity of the bone marrow cells for all groups of the present experiments, the mitotic frequencies were significantly decreased in intoxicated groups either untreated or treated with camel milk. This parameter was increased and ameliorated by the camel milk treatments but was still lower than the normal healthy controls (G1 and G2).

### 3.3 Histological examination

**FIGURE 1:**
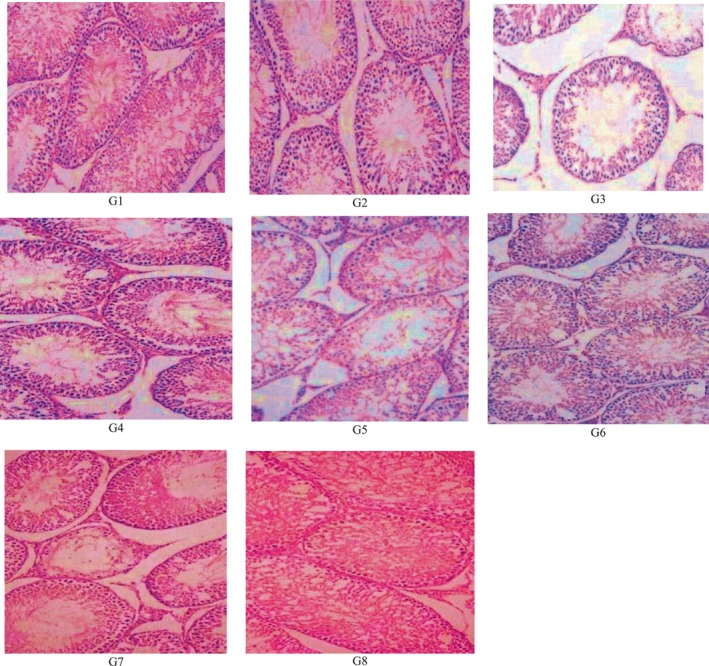
Histopathological structure of testis tissues of male albino rats. (a) Normal healthy control (G1). (b) Normal healthy rats treated with camel milk (G2). (c) Fipronil intoxicated control (G3). (d) Fipronil intoxicated rats treated with camel milk (G4). (e) Lead acetate intoxicated control (G5). (f) Lead acetate intoxicated rats treated with camel milk (G6). (g) Lead + fipronil mixture intoxicated control (G7). (h) Lead + fipronil mixture intoxicated rats treated with camel milk (G8).

For the histological examination, the testis microscopy of either of the normal healthy control groups (G1 and G2) or groups treated with camel milk (G4, G6, and G8) showed a normal histopathological structure with spermatogenic cells lining the seminiferous tubules in several layers, which are presented in Figure 1. For the group (G3) that ingested fipronil, there was mild widespread degeneration in the seminiferous tubules, decreases in the thickness of lining epithelium, and vacuolation of Sertoli cells. In the treatment with camel milk for the fipronil intoxicated group (G4), the seminiferous tubules in the testis microscopy have minimal degradation in the intoxicated lead control animals (G5), reduced spermatozoa were also observed in the lumen of several seminiferous tubules, along with desquamated spermatocytes and early spermatids. Rats with lead intoxicated were treated with camel milk (G6) showing that the testicular lesions were partially ameliorated relative to its intoxicated control group (G5). In the fipronil + lead mixture intoxicated control group (G7), the results shown in testicular microscopy revealed a weakening of the germinal epithelium and significant widespread degeneration in the seminiferous tubules. Seminiferous tubules also demonstrated intraluminal infiltration of homogenous hyalinized eosinophilic material and a moderate to severe reduction in luminal spermatozoa. The camel milk‐treated group against the toxicity of lead + fipronil mixture induction (G8) showed decreases in degeneration of seminiferous tubules relative to those of its intoxicated animal control (G7). The results indicate, according to Johnsen's score, that spermatogenesis was impaired in the three intoxicated control groups G3, G5, and G7. This impairment was improved and partially restored by the treatment with camel milk in groups G4, G6, and G8, but the reductions were still recorded in comparison with the normal healthy control groups G1 and G2. Also, the three intoxicated control groups had less epithelial thickness lining seminiferous tubules (G3, G5, and G7) which were improved in groups by treatments with camel milk (G4, G6, and G8).

Also, other studies documented the disorders of reproduction due to lead acetate and fipronil as well as their mixture and the mutual influence of the two xenobiotics (Ibrahim et al., 2012; Noaishi et al., 2021). For that, the present work studied the mutual deleterious effect of lead acetate and fipronil on liver oxidative stress, spermatogenesis, chromosomal aberrations, and testicular histopathology in male albino rats. Also, the study measured the possible improvement and protective influences of camel milk against the toxic harmful of lead and fipronil.

In the present work, fipronil and lead acetate exposure produced significant increase in the oxidative stress which concurs with Noaishi et al. (2021) who reported that oxidative stress is one of the causative factors which links abnormal function and dyslipidemia (Abdel‐Rahim et al., 2014; Ibrahim et al., 2012; Kandil et al., 2020; Noaishi et al., 2021). Fipronil also elevated the oxidative stress of male albino rats. SOD, CAT, and GST activities significantly decreased compared to normal healthy control. MDA was significantly increased under the exposure of fipronil (Noaishi et al., 2020). Lead has different effects on the immune system depending on the dose and duration, whether they are stimulatory or suppressive (Ibrahim et al., 2012). The lead acetate exposure had the same trend as fipronil for MDA, SOD, CAT, and GST as antioxidative system. These are in agreement with Ibrahim et al. (2012) and Abdel‐Rahim et al. (2014). A critical balance exists between the rate of hydrogen peroxide production by oxygen dismutation by SOD activity and the rate of hydrogen peroxide elimination by CAT activity under normal physiological conditions. However, the impairment in the present pathway will influence the activities of other antioxidative enzyme system in the animal (Kon & Fridorich, 1992). Lead and fipronil, as well as their combination, can easily enter the cell membrane and cause lipid peroxidation of the membrane, which may cause the produced oxidative stress. The treatment with camel milk against the above xenobiotics as antioxidative agent ameliorates the influences of either lead or fipronil toxicity by scavenging and neutralizing the ROS (reactive oxygen species) (Abdel‐Mobdy et al., 2023).

The count of sperm abnormalities, sperm count, and testicular relative weight was influenced by lead acetate and fipronil and their mixture in the present investigations under the oxidative stress conditions similar to other research (Abdel‐Mobdy, 2021). The abnormalities of the sperm result in a point of mutation in germ cells (Narayana et al., 2002) which could have influenced the normal spermatogenesis (Abdel‐Aziem et al., 2005).

The chromosomal aberrations were significantly elevated in lead acetate and fipronil and their mixture exposures. The mutagenic influences of lead and fipronil were found, as that both induced chromosomal aberration due to a possible clastogenic influence under oxidative stress (Abdel‐Mobdy et al., 2021). Both xenobiotics reduced the mitotic frequencies inferring their cytotoxic influences.

Histopathological examination of the testis tissue showed degeneration and desquamation of spermatogenic cells lining seminiferous tubules with complete absence of spermatozoa in some seminiferous tubules, confirming the reproductive dysfunction exhibited by lead and fipronil exposures. Testicular degeneration was marked in the animals induced lead and fipronil. Similar investigations reported the reproductive disorders associated with oxidative stress of ϒ‐irradiation in male albino rats (Abdel‐Mobdy et al., 2021). Antioxidant camel milk ingestion can alleviate the harmful toxic effects of lead and fipronil on the sperm shape and chromosomal aberrations in male albino rats likewise to the influences of ϒ‐irradiation (Abdel‐Mobdy et al., 2021). Camel milk treatments against the toxicity of lead and fipronil attenuated the reproductive disorders, peroxidation of lipids, histopathological examination, and oxidative stress caused by lead acetate and their mixture. These beneficial influences of camel milk could be explained by the high contents of vitamins, proteins, enzymes, minerals, amino acids, and different antioxidants etc(Abdel‐Mobdy et al., 2023; Gad et al., 2011).

Generally, it can be concluded that lead and fipronil exposures induced oxidative stress, increased sperm abnormalities, and decreased sperm count with the increases in chromosomal aberrations and histopathological variation in testis tissue, which were enhanced by the use of camel milk treatments. Histopathological and biochemical analyses confirmed each other. Also, this indicates that camel milk treatments possess an antioxidative activity, making camel milk a promising factor as a protective agent against xenobiotic toxicity and harm produced by oxidative stress.

## 4 CONCLUSIONS

Camel milk contains casein which is the major protein with a higher value. High levels of cysteine in the whey protein of camel milk are responsible for increasing GSH production, which in turn reduces the amount of free radical scavenging caused by exposure to xenobiotics. The biological effects of camel milk antioxidative agent included immunological activation, changes in metabolic processes, and anticytotoxic activity in animals. Additionally, these agents can prevent genetic alterations by reducing the damage to DNA, proteins, membranes, and fats by ROS. For the current toxicant exposure, the toxic effects of lead and fipronil exposure on the oxidative system, mutagenicity, and fertility of male albino rats can be ranked as follows in decreasing order: lead + fipronil mixture > fipronil > lead acetate.

## AUTHOR CONTRIBUTIONS

**Yasmin E. Abdel‐Mobdy:** Writing – original draft (equal). **Ahmed E. Abdel‐Mobdy:** Writing – review and editing (equal).

## FUNDING INFORMATION

This research did not receive any specific grant from funding agencies in the public, commercial, or nonprofit sectors.

## CONFLICT OF INTEREST STATEMENT

The authors have no conflict of interest to declare.

## ETHICS STATEMENT

All ethical principles were considered in this article.

## CONSENT TO PARTICIPATE

All have participated and approved the manuscript and agreed with submission to Environmental Science and Pollution Research.

## CONSENT TO PUBLISH

This manuscript has not been published elsewhere and is not under consideration by another journal. We believe that the findings of this study are relevant to the scope of your journal and will be of interest to its readership.

## Data Availability

Samples of the compounds and data used during the current study are available from the corresponding author.
